# 表观遗传学在恶性肿瘤发生发展和治疗中的新进展

**DOI:** 10.3779/j.issn.1009-3419.2020.02.04

**Published:** 2020-02-20

**Authors:** 攀 王, 洪林 赵, 凡 任, 青春 赵, 睿峰 施, 兴雨 刘, 京豪 刘, 永文 李, 颖 李, 红雨 刘, 军 陈

**Affiliations:** 1 300052 天津，天津医科大学总医院肺部肿瘤外科 Department of Lung Cancer Surgery, Tianjin Medical University General Hospital, Tianjin Lung Cancer Institute, Tianjin Key Laboratory of lung Cancer Metastasis and Tumor Microenvironment, Tianjin 300052, China; 2 300052 天津，天津市肺癌研究所，天津市肺癌转移与肿瘤微环境重点实验室

**Keywords:** 肿瘤, 表观遗传学, 表观遗传治疗, 联合用药, Neoplasms, Epigenomics, Epigenetic therapy, Drug combinations

## Abstract

表观遗传学修饰与肿瘤的发生发展密切相关，其主要通过DNA甲基化、组蛋白修饰、非编码RNA调控和染色质结构重构等方式对基因功能和表达水平进行调控，从而影响肿瘤的进展。目前针对表观遗传学的药物已经逐渐应用于恶性肿瘤的治疗，常见的药物类型包括DNA甲基转移酶抑制剂和组蛋白去乙酰化酶抑制剂，但此类药物仍存在诸多不足之处广泛的临床应用仍需要进一步的研究，令人鼓舞的是表观遗传药物与多种抗肿瘤药物联合应用已表现出巨大的应用潜力。本文就表观遗传学在恶性肿瘤的发生发展机制和相关药物的新进展进行了综述。

## Abstract (en)

Epigenetic modification is closely related to the occurrence and development of tumors. It mainly regulates gene function and expression level through DNA methylation, histone modification, regulation of non-coding RNA and chromatin structure reconstruction. At present, epigenetic drugs have been gradually applied to the treatment of malignant tumors. Common drug types include: DNA methyltransferase inhibitors and histone deacetylase inhibitors. However, these drugs still have many shortcomings and a wide range of clinical applications need further research. Encouragingly, the epigenetic drugs in combination with various anti-tumor drugs have shown great application potential. In this paper, we summarized the development mechanism of epigenetics in malignant tumors and the progress of related drugs.

在全球范围内，肺癌的发病率和死亡率仍占恶性肿瘤的首位，预计2018年将新增肺癌患者210万人，将有180万人因肺癌死亡，约占癌症死亡人数的18.4%^[1]^。在大多数类型的癌症中基因改变的频率通常很低^[2, 3]^，但是在人类几乎所有癌症种类中表观遗传学的改变远远超过体细胞的基因突变^[4-6]^，表观遗传学是除DNA序列之外影响基因表达和细胞表型的重要因素，其影响因素包括在胞嘧啶残基上的DNA甲基化、在组蛋白尾上添加乙酰基和甲基、非编码RNA表达和染色质结构重构等^[7]^，特别是DNA甲基化的研究较早且进展显著。此外，因为表观遗传具有可逆性的特点，所以针对表观遗传的药物为恶性肿瘤的治疗提供了新的方向和策略，目前常见的药物类型包括DNA甲基转移酶抑制剂和组蛋白去乙酰化酶抑制剂，并且表观遗传药物与多种抗肿瘤药物联合应用也具有广泛的应用前景。本文就表观遗传学在恶性肿瘤发生发展和治疗中的新进展进行综述。

## 1 表观遗传学基础和在肿瘤中异常表现

表观遗传学的主要研究内容包括：DNA甲基化、组蛋白修饰、非编码RNA调控和染色质结构重构。表观遗传的修饰在不改变DNA序列的同时能调控基因的表达和（或）转录从而影响胚胎发育、干细胞的分化、衰老和肿瘤发生等过程^[8]^。

### 1.1 DNA甲基化

DNA甲基化是指在甲基转移酶（DNMT1、DNMT3A和DNMT3B）的催化下，将S腺苷甲硫氨酸（S-adenosylmethionine, SAM）提供的甲基转移到胞嘧啶5位的碳原子，形成5′甲基胞嘧啶^[9]^。DNMT1主要是维持DNA甲基化，通过DNA复制将亲代甲基化DNA遗传到子代DNA。DNMT3A和DNMT3B能将未甲基化的CpG位点甲基化，这在胚胎发育和肿瘤发生中起着重要作用。DNMT2是一种tRNA甲基转移酶，而DNMT3L是在胚胎发育过程中协调DNMT3A和DNMT3B的结构蛋白^[10]^。DNMT3C最近由Barau等^[11]^发现，主要涉及雄性生殖细胞系的反转录转座子启动子的甲基化。在肿瘤的发生过程中，启动子CpG岛胞嘧啶的异常高度甲基化和整体基因的低甲基化导致整个基因组的不稳定和基因表达谱改变，包括抑癌基因，内源性逆转录酶病毒和肿瘤抗原的沉默以及癌基因的表达上调^[12-15]^。最新研究表明肺癌患者不同强度的吸烟习惯与CpG位点基因甲基化有很强的相关性^[16]^。除肿瘤以外，DNA甲基化还与衰老相关性疾病，精神疾病和免疫系统疾病等相关^[17]^。肿瘤特异性甲基化的基因能在循环肿瘤细胞（circulating tumor cells, CTCs）、血液、尿液及其他体液中被检测，因此常用于早期肿瘤的诊断和预后的研究。在结肠、前列腺、乳腺、肝脏肿瘤和白血病中也发现DNMT3A、DNMT3B和DNMT3L的含量常常升高^[18-23]^。研究表明DNMT3A的缺乏会引起部分基因的低甲基化并抑制白血病的转化^[24]^，但也有证据表明DNMT3A和DNMT3B是抑制淋巴系统恶性肿瘤的肿瘤抑制基因^[25]^。

### 1.2 组蛋白修饰

组蛋白是由H1、H3、H2A、H2B和H4这5种类型的核心蛋白组成的高度保守的蛋白质，并与DNA共同构成核小体。组蛋白氨基末端会在翻译后被修饰，组蛋白修饰后的类型和DNA甲基化的程度将会决定特定的染色质结构^[26]^。组蛋白修饰包括磷酸化、甲基化、乙酰化、泛素化、糖基化、ADP核糖基化、去氨基化、类泛素化和脯氨酸异构化等^[27]^。组蛋白尾部残端不同的修饰类型与特定蛋白相互作用，从而将染色质分为异染色质和常染色质^[26]^。组蛋白修饰不仅可逆性抑制或促进基因转录而且还可以影响DNA修复、复制、干细胞形成和细胞状态变化等过程^[28]^。研究表明H3K9ac、H3K9me3、H4K16ac水平降低与非小细胞肺癌和急性髓系白血病的复发相关^[29]^，组蛋白H3K4ac、H3K18ac和H3K27me3的修饰在口腔鳞癌的进展和预后中起重要作用，组蛋白甲基转移酶Suv39h、Ezh2、MLL、Nsd1、Riz与肿瘤相关，其中Ezh2过表达与乳腺癌、前列腺癌、多发性骨髓瘤和淋巴瘤密切相关^[30]^。

### 1.3 非编码RNA调控

非编码RNA（non-coding RNA, ncRNA）是指能被转录但不能翻译为蛋白质的功能性RNA，主要包括IncRNA、miRNA、circRNA、rRNA、tRNA、snRNA、snoRNA等。IncRNA参与许多重要生物学现象，例如印记基因组位点，形成染色体构象和变构调节酶活性^[31, 32]^。IncRNA表达的特定模式能影响细胞状态、分化、发育和疾病^[33]^。miRNA不仅能在转录后水平抑制靶基因表达或诱导其降解和翻译抑制，而且在转录水平上与靶基因启动子形成互补序列从而导致基因沉默^[34]^，因此miRNA可诱导靶mRNA的双重抑制。circRNA由pre-mRNA中的外显子反向剪切形成的单链环状RNA分子，circRNA可以通过滴定microRNA来调节转录和干扰剪切进一步影响基因表达^[35]^。一些circRNA能调控重要的肿瘤特性，包括肿瘤细胞的增殖、凋亡、转移、代谢、衰老和耐药性甚至与肿瘤患者的预后相关^[36-39]^。circRNA通常为低水平表达^[40]^，但最新研究发现数十种circRNA以细胞或组织特异性的方式高度表达^[41, 42]^。尽管大多数环状RNA的功能尚不清楚，但其在基因调控中的重要作用正在被逐步证实^[35, 43, 44]^。最新的研究表明部分非编码RNA也能编码多肽，例如植物中的蒺藜状苜蓿的pri-miR171b和拟南芥的pri-miR165a能产生多肽，这些多肽能增强相应的成熟miRNAs的产生，从而下调参与根部发育的靶基因的表达^[45]^。

### 1.4 染色质结构重构

在DNA转录时染色质由紧密的超螺旋结构变构为开放式的疏松结构，这种不改变DNA碱基序列的结构改变称为染色质重塑。染色质重构与基因表达、凋亡、DNA复制和修复、以及肿瘤的发生密切相关^[46]^。其主要机制包括：ATP依赖的染色质重塑复合物、共价组蛋白修饰、组蛋白变异和DNA甲基化^[47]^。根据功能结构域的不同可以将ATP依赖的重塑复合物主要分为ISWI、SWI/SNF、INO80和CHD等亚家族^[48, 49]^。ISWI复合物在核小体阵列和核小体自由区重塑从而调控基因表达^[50]^、异染色质的建立与复制^[51]^、DNA修复^[52]^以及rRNA基因表达的协调^[53]^。研究表明SWI/SNF家族复合物是肿瘤抑制因子而非致癌因子^[54, 55]^，SWI/SNF家族复合物是在特定组织中产生特殊功能的广泛多样性的复合物。在对44项全基因组和外显子组测序报道的人类肿瘤中，哺乳动物的SWI/SNF复合物的大多数突变高于背景突变率，其突变率达到19.6%，广泛涉及实体和血液系统肿瘤，包括：卵巢透明细胞、胰腺、肾细胞、肝细胞、膀胱、胃、乳腺和血液恶性肿瘤，甚至在某些癌症中不止一个亚基突变^[56]^。INO80复合物能促进DNA复制叉的稳定、DNA合成的恢复和DNA损伤的耐受，这些与复制叉上重构核小体有关。INO80复合物还参与端粒调节、着丝粒稳定性和染色体分离^[57]^。研究发现在前列腺癌中TP53位点的缺失与局部三维结构的改变和新的拓扑相关域边界形成相关^[58]^。

## 2 表观遗传药物治疗进展

与肿瘤的遗传因素相比，可逆的表观遗传变异可以通过化学药物调节，在癌症治疗中具有广泛的前景。目前针对表观遗传学的药物正在逐步被开发并用于肿瘤的治疗。主要包括：DNA甲基转移酶抑制剂、组蛋白去乙酰化酶抑制剂和联合用药。

### 2.1 DNA甲基转移酶抑制剂（DNA methyltransferase inhibitors, DNMTis）

核苷类抑制剂：通过抑制DNMTs的活性来促进抑癌基因的表达从而减少肿瘤细胞的产生，所以DNMTs可以作为特异性抗肿瘤药物的有效靶点^[59]^，其主要包括核苷类和非核苷类^[60]^。核苷类抑制剂主要包括azacitidine（AZA）和decitabine。低剂量的azacitidine和decitabine可以诱导之前因甲基化而沉默的细胞周期蛋白再次活化从而诱导细胞分化，减少增殖和增加子代细胞的凋亡，高剂量用药可直接引起肿瘤细胞死亡^[61, 62]^。低剂量的DNMTi能够在避免细胞毒性的同时实现全基因组的DNA甲基化和转录组的持续变化^[63]^。Azacitidine主要是通过核苷转运体进入哺乳动物的DNA和RNA中发挥作用，与RNA相比其与DNA结合程度较低，但其对DNA的合成抑制作用远大于对RNA。而decitabine可被不同的激酶磷酸化并且仅存在于DNA中^[64]^。Azacitidine通过非竞争性抑制DNA甲基转移酶（DNA methyltransferase 1, DNMT1）使胞嘧啶甲基化阻滞，从而导致甲基转移酶耗尽实现DNA的低甲基化，但对静止的不能分裂的细胞无效^[65, 66]^。AZA最初用于治疗急性髓系白血病患者，在发现其在甲基化机制中的作用后被美国食品药品监督管理局（Food and Drug Administration, FDA）批准用于治疗骨髓增生异常综合征（Myelodysplastic Syndromes, MDS）的一线药物^[67]^。Decitabine对肿瘤有双重作用，在高剂量是具有细胞毒性而低剂量时可有效绕过突变性凋亡缺陷^[68-70]^。在azacytidine对胃肠癌、肺癌、乳腺癌和黑色素瘤等实体肿瘤的研究中发现最好的用药效果往往不是使用最大量或者最大的耐受剂量^[71, 72]^。考虑到azacitidine和decitabine的作用机制略有不同以及所涉及的不同的代谢活化途径，两种低甲基化剂之间不一定存在完全的交叉抗性。一种药物的失败可能不排除另一种药物的疗效^[73]^。Azacitidine和decitabine现已被FDA批准用于MDS和白血病的治疗^[74]^，其常见的副作用包括恶心、呕吐、腹泻甚至致突变损伤^[71]^。现有的核苷类DNMT抑制剂的不足之处主要包括口服生物利用度低，稳定性差，而且药物毒副作用大，最常见的毒性反应是骨髓移植，主要表现为中性粒细胞减少症和血小板减少症，因此限制了其在恶性肿瘤中的应用^[75-77]^。

核苷衍生物抑制剂：与恶性血液病相比，治疗实体瘤的难题之一就是实体瘤分裂细胞数量有限而且azacitidine和decitabine是S期特异性药物需要结合到DNA才能实现其表观遗传效应，另外这两种抑制剂的氮杂胞嘧啶环在水溶液中非常不稳定，甚至因对血清中的胞苷脱氨酶更敏感从而限制了其应用。既往研究发现在顺铂联合decitabine治疗非小细胞肺癌患者并不比单用顺铂有明显改善，预估其客观疗效为15%，联合用药患者的生存期未超过1年，低于其他含顺铂的联合治疗^[78]^，因此更稳定的下一代甲基化抑制剂例如zebularine、S-110和NPEOC-DAC有望提高对肿瘤的疗效^[71]^。相比于核苷类，核苷衍生物抑制剂例如zebularine则具有更高的稳定性和更低的毒性^[79]^。Zebularine是一种缺乏4-氨基的胞苷，其不仅可以抑制DNA甲基化并重新激活沉默基因而且可以增强肿瘤细胞对化疗和放疗的敏感性、抑制细胞分裂和血管活性，其作用机制是通过稳定DNMTs与DNA的结合来阻止甲基化和抑制复合物解离并且其细胞毒性低所以可以长时间用药维持去甲基化的状态^[80]^。此外，胞苷类似物需要转化成5-aza-2脱氧氮杂胞苷三磷酸才能与DNA结合而且是通过抑制甲基化的酶来调控新的细胞分裂从而稀释甲基化的DNA^[81]^，这就部分解释了为何甲基化抑制剂的临床效果常常有滞后性^[82]^加上其半衰期短，所以对于那些增殖性低的疾病可能会影响用药效果^[83]^。S-110是一种双核苷酸，由5-aza-2′-脱氧胞苷和脱氧尿苷组成。可诱导p16表达并且抑制胞苷脱氧酶的脱氨作用而比5-aza-2′-脱氧胞苷更稳定，是一种耐受性好、毒性小的新型药物^[84]^。

非核苷类DNMT抑制剂：非核苷类DNMT抑制剂在白血病，前列腺和乳腺癌中表现出良好的抗肿瘤作用^[60, 85]^。使用硅片筛选方法和甲基化抑制试验确定出一种新型的选择性非核苷DNMT1抑制剂DC_05能显著抑制癌细胞增殖^[86]^。SGI-1027是一种喹诺酮类非核苷化合物，能快速诱导多种癌细胞中DNMT1蛋白酶的降解并诱导沉默的抑癌基因P16、MLH1和TIMP3的去甲基化和重新表达^[87]^。理论上讲长度为4个-8个核苷酸的RNA分子可以充分地占据DNMT的催化区域而且不超过30个核苷酸的RNA使用更方便，因此针对DNMTs的小RNA抑制剂具有很好的应用前景。RNA抑制剂可以通过竞争性抑制来降低DNMT1的活性，miR-155-5p在人结直肠癌细胞系HCT-116中可明显增加基因组的低甲基化，免疫共沉淀实验进一步证实了miR-155-5p能与HCT细胞中的DNMT1结合从而抑制酶的活性^[86]^。小干扰RNA是一种具有显著基因调控的效率的非编码RNA，能有效治疗感染和癌症等多种疾病^[88]^，siRNA通过转录后水平激活RNA诱导的沉默复合物直接诱导靶基因沉默。由腺病毒复制表达的干扰性长链非编码RNAs（long noncoding RNAs, lncRNAs）与肿瘤相关的内源性microRNAs oncogenic miRNAs（OncomiR）的靶基因竞争性结合OncomiR并消耗OncomiRs，从而保护大量抑癌基因不被OncomiRs抑制，从而实现对癌细胞的靶向干预治疗^[89]^。还有针对DNMT3A的小分子抑制剂5-azacytidine已经被批准用于急性髓细胞性白血病（Acute myeloid leukemia, AML）患者的临床治疗^[90]^。DOT1L抑制剂SYC-52221和EPZ004777以剂量和时间依赖的方式减少DNMT3A突变细胞系肿瘤细胞增殖并诱导细胞凋亡、周期阻滞和终末分化^[91]^。

### 2.2 组蛋白去乙酰化酶抑制剂（histone deacetylase inhibitor, HDACi）

组蛋白去乙酰化酶是一组从组蛋白赖氨酸尾部去除乙酰基的高度保守的酶^[92]^。HDAC对组蛋白的去乙酰化促进了染色质的闭合和抑制基因转录。此外非组蛋白的乙酰化也非常重要^[93, 94]^。因此乙酰化和去乙酰化共同维持基因的正常转录^[95]^。HDACi是一种新的抗肿瘤药物，通过调节基因表达发挥其作用。其对恶性肿瘤有广泛的作用，主要包括抑制细胞分化、抑制细胞周期生长、抑制血管生成、细胞凋亡和免疫调节^[96, 97]^。HDACi在各种血液系统恶性肿瘤都用肿瘤抑制作用，包括：霍奇金淋巴瘤、骨髓恶性肿瘤、皮肤T细胞淋巴瘤和外周T细胞淋巴瘤^[98, 99]^。HDACi通常耐受性较好，最常见的副作用是疲劳，胃肠道紊乱和可逆性骨髓抑制，其中大部分为轻度至中度^[98]^，而且不增加心脏毒性^[100]^。现在vorinostat和romidepsin在美国已经被批准用于皮肤T细胞淋巴瘤的治疗。在动物模型中研究发现HDAC抑制剂通过下调正细胞周期调节因子如细胞周期蛋白D1、c-Myc和AKT来重建黑色素瘤细胞的凋亡、诱导细胞分化、抑制肿瘤生长和诱导抗恶性肿瘤增殖的基因表达^[101-104]^。HDAC抑制剂通过阻断关键性黑色素瘤细胞激活酶从而抑制活化的MEK1/2和ERK1/2的表达^[105]^。此外，HDAC抑制剂同时可能干扰了对癌细胞生长至关重要的HSP90-client蛋白（包括AKT和RAF）的有效折叠^[102, 106]^。但是，HDAC抑制剂不能重新激活因启动子甲基化而导致的沉默基因的表达，这也为在HDAC抑制剂使用后再运用DNMT抑制剂提供了理论依据，两种药通过协同作用增强基因的重新表达和药物敏感性^[107]^。此外，小分子抑制剂包括针对组蛋白去乙酰化酶（histone deacetylases, HDACs）的belinostat和针对组蛋白甲基转移酶EZH2的抑制剂EPZ6438已被批准用于临床试验^[90]^。EZH2抑制剂EPZ005687对EZH2有高选择性，从而显着降低了带有EZH2 Y641和A677突变的淋巴瘤细胞系的生存能力^[108]^。

### 2.3 联合用药

研究发现表观遗传沉默可能导致肿瘤对化疗药物的抵抗，而针对表观遗传机制的药物会增强对化疗的敏感性^[109, 110]^，而且多项研究表明表观遗传药物似乎可以通过几种机制增强内源性抗肿瘤免疫应答^[111-113]^，其中DNA去甲基化是克服肿瘤免疫逃逸的有效途径^[114]^。DNA甲基化抑制剂和HDAC抑制剂的联合用药正在进行临床试验^[115-117]^，此外还有与维甲酸、免疫调节剂和酪氨酸激酶抑制剂联合用药治疗血液系统恶性肿瘤^[118]^。在探索DNA甲基转移酶抑制剂decitabine和组蛋白去乙酰化酶抑制剂panobinostat联合烷化剂temozolomide治疗黑色素瘤安全性和耐受性的Ⅱ期临床实验证明联合用药是安全可行的^[104]^。一些研究表明，经p21介导的生长停滞的细胞在低甲基化剂和组蛋白去乙酰酶抑制剂（histone deacetylase inhibitor, HDI）的细胞毒性作用下解除抑制^[119, 120]^。实体瘤和恶性血液病对HDAC抑制剂的敏感性不同的一个可能的原因就是后者具有较少的基因突变和完整的凋亡途径。在对11例乳腺癌和11例结直肠癌测序的13, 023个基因中，单个肿瘤平均有90个突变基因，其中11个为致癌基因，而且在实体瘤中的大量突变基因通常在不同肿瘤中是不重叠的^[121]^。如果实体瘤具有内在的耐药机制，那么增加剂量或者开发针对于HDI分子效应的药物可能会克服其耐药性。一项Ⅱ期研究表明azacitidine（每天75 mg/m^2^，连续5 d）和免疫调节药物lenalidomide（10 mg/d，21 d，28 d为1个周期）的联合用药患者的耐受性良好，并且对高风险MDS的患者非常有效，44%的患者达到完全缓解，总反应率为72%^[122]^。HDACi联合Aza可以通过增强免疫信号和减少MYC驱动的细胞增殖从而实现强大的抗肿瘤作用。此外，组蛋白脱乙酰基酶抑制剂可以增强PD-1免疫治疗在肺腺癌和黑色素瘤的的疗效^[123]^。随着系统生物学的发展，DNMTi与HDACi或免疫治疗相结合可能成为表观遗传治疗的一种有效途径。

另外有研究表明合理调节染色体密度波动可以导致癌细胞的整体转录活性和细胞间转录异质性的降低，从而在体外实现近乎完全的癌细胞杀伤。因此调节染色质的物理空间结构可以改变基因表达情况从而实现抗肿瘤作用^[124]^。

## 3 展望

目前，针对表观遗传学的药物并不能广泛用于肿瘤的治疗而且疗效并不十分理想，很多药物存在生物利用率低、稳定性差和毒性大以及用药时间和剂量的不规范等不足之处，令人鼓舞的是针对这些问题已经找到部分解决方案并且已有很多改进型药物已进入临床研究阶段，而且诸多研究已经提示不同类型的表观遗传学药物联合用药以及与其他化疗、免疫治疗等药物联用具有广泛的抗肿瘤治疗前景，这也将会为肿瘤的治疗开辟一条新的途径。

甲基化抑制剂在水溶液中很不稳定，能自然水解并可在胞苷脱氨酶（cytidine deaminase, CDA）的介导下脱氨水解从而限制了其在实体瘤中的应用^[73]^，而且CDA在肠道和肝脏中高度表达，因此限制了其口服制剂的应用，临床上也常常静脉或者皮下等肠外途径给药，如果与CDA抑制剂联合用药可以延长肿瘤细胞在低甲基化药物（hypomethylating agent, HMA）中的暴露时间从而改善治疗效果。四氢尿苷（tetrahydrouridine, THU）是胞苷脱氨酶的竞争性抑制剂，在狒狒和小鼠模型中，口服THU可显著提高decitabine的口服生物利用度，产生合适的消耗DNMT1的浓度-时间曲线，并降低了个体间的变异性。因此口服THU-DAC制剂可能有助于获得安全有效的DNMT1靶向治疗^[125]^。

Azacitidine和decitabine是S期特异性药物，从理论上讲，长时间的药物暴露会影响更多的细胞并且提高疗效，但是随着剂量的增加和接触时间的延长，在细胞毒性和去甲基化的双重作用下，骨髓抑制变得更严重，需要数周才能恢复。因此，相比与使用最大耐受剂量，找到骨髓抑制和需长期暴露才能获得抗肿瘤效果之间的最佳平衡剂量才是临床用药的核心问题^[126]^。另外，在骨髓抑制的恢复过程中甲基化会再次发生^[69, 127]^，尚不清楚是去甲基化的细胞再次甲基化还是短期暴露于去甲基化药物的未增殖细胞再次增殖同时去甲基化的细胞已经终止分化成为静止状态或者死亡。

甲基化抑制剂能在血液系统中成功应用，而在实体瘤中疗效有限，一个可能的原因就是早期研究中使用的药物剂量通常是最大耐受剂量，最终使DNA合成抑制，而非抑制DNMT和甲基化^[62, 128]^。另一个可能的原因就是，实现低甲基化所需的药物剂量远低于通常的细胞毒作用的剂量^[68, 129]^，因为需要活跃的细胞周期来实现甲基化逆转，因此延长用药疗程可能比短期疗程能实现更多的去甲基化。在骨髓瘤中，延长低剂量的甲基化抑制剂可能会提高或维持肿瘤对药物的应答率^[130]^。因此当已经实现和维持甲基化逆转时，保持长期的低剂量维持治疗是关键，这比为达到剂量限制性毒性或最大耐受剂量而提高药物的剂量可能更为有效^[131]^。大多数用decitabine治疗实体瘤时采用高致毒性的剂量和短疗程，这可能是与血液系统恶性肿瘤相比在实体瘤中临床效果不佳的原因之一^[132, 133]^。

最新研究认为短期内应用表观遗传学治疗能诱导活化的效应T细胞的聚集，而长期应用则可以诱导衰竭的T细胞表型的改变形成高效的效应T细胞。这种记忆细胞表型的获得与肿瘤内激活的T细胞的积累为免疫治疗靶点的长期有效性提供了基础，也为表观遗传学疗法与免疫治疗的联合用药理论支撑。组蛋白去甲基化酶1（lysine specific demethylase 1, LSD1）在调节双链RNA（double-stranded RNA, dsRNA）和干扰素方面起着关键作用，LSD1的表达与CD8^+^ T细胞浸润及预后呈负相关，抑制LSD1可以有效逆转对程序性死亡分子1（programmed death-1, PD-1）耐药的肿瘤细胞从而提高PD-1的疗效，提示靶向LSD1抑制剂联合PD-L1（programmed cell death-Ligand 1）阻断剂治疗癌症的潜力^[134]^。另外也希望在不远的将来能发现参与染色质重塑的酶从而生产出新型的表观遗传调节剂。总之，随着表观遗传学研究进一步深入，开发针对肿瘤的表观遗传药物将具有巨大的应用前景。

## References

[1] Bray F, Ferlay J, Soerjomataram I (2018). Global cancer statistics 2018: GLOBOCAN estimates of incidence and mortality worldwide for 36 cancers in 185 countries. CA Cancer J Clin.

[2] Prasad V (2016). Perspective: the precision-oncology illusion. Nature.

[3] Klauschen F, Andreeff M, Keilholz U (2014). The combinatorial complexity of cancer precision medicine. Oncoscience.

[4] Hoadley KA, Yau C, Wolf DM (2014). Multiplatform analysis of 12 cancer types reveals molecular classification within and across tissues of origin. Cell.

[5] Witte T, Plass C, Gerhauser C Pan-cancer patterns of DNA methylation. Genome Med.

[6] Schuebel KE, Chen W, Cope L (2007). Comparing the DNA hypermethylome with gene mutations in human colorectal cancer. PLoS Genet.

[7] Werner RJ, Kelly AD, Issa JJ Epigenetics and precision oncology. Cancer J.

[8] Mazzio EA, Soliman KF (2012). Basic concepts of epigenetics: impact of environmental signals on gene expression. Epigenetics.

[9] Howell PM, Liu S, Ren S (2009). Epigenetics in human melanoma. Cancer Control.

[10] Jones PA, Liang G (2009). Rethinking how DNA methylation patterns are maintained. Nat Rev Genet.

[11] Barau J, Teissandier A, Zamudio N (2016). The DNA methyltransferase DNMT3C protects male germ cells from transposon activity. Science.

[12] Baer C, Claus R, Plass C (2013). Genome-wide epigenetic regulation of miRNAs in cancer. Cancer Res.

[13] Liang G, Weisenberger DJ (2017). DNA methylation aberrancies as a guide for surveillance and treatment of human cancers. Epigenetics.

[14] Yoo CB, Jones PA (2006). Epigenetic therapy of cancer: past, present and future. Nat Rev Drug Discov.

[15] Baylin SB, Jones PA (2011). A decade of exploring the cancer epigenome-biological and translational implications. Nat Rev Cancer.

[16] Ma B, Huang Z, Wang Q (2019). Integrative analysis of genetic and epigenetic profiling of lung squamous cell carcinoma (LSCC) patients to identify smoking level relevant biomarkers. BioData Min.

[17] Erdmann A, Halby L, Fahy J (2015). Targeting DNA methylation with small molecules: what's next?. J Med Chem.

[18] el-Deiry WS, Nelkin BD, Celano P (1991). High expression of the DNA methyltransferase gene characterizes human neoplastic cells and progression stages of colon cancer. Proc Natl Acad Sci U S A.

[19] Patra SK, Patra A, Zhao H (2002). DNA methyltransferase and demethylase in human prostate cancer. Mol Carcinog.

[20] Girault I, Tozlu S, Lidereau R (2003). Expression analysis of DNA methyltransferases 1, 3A, and 3B in sporadic breast carcinomas. Clin Cancer Res.

[21] Girault I, Lerebours F, Amarir S (2003). Expression analysis of estrogen receptor alpha coregulators in breast carcinoma: evidence that NCOR1 expression is predictive of the response to tamoxifen. Clin Cancer Res.

[22] Oh BK, Kim H, Park HJ (2007). DNA methyltransferase expression and DNA methylation in human hepatocellular carcinoma and their clinicopathological correlation. Int J Mol Med.

[23] Melki JR, Warnecke P, Vincent PC (1998). Increased DNA methyltransferase expression in leukaemia. Leukemia.

[24] Yang L, Rodriguez B, Mayle A (2016). DNMT3A loss drives enhancer hypomethylation in FLT3-ITD-associated leukemias. Cancer Cell.

[25] Peters SL, Hlady RA, Opavska J (2014). Tumor suppressor functions of Dnmt3a and Dnmt3b in the prevention of malignant mouse lymphopoiesis. Leukemia.

[26] Misri S, Pandita S, Kumar R (2008). Telomeres, histone code, and DNA damage response. Cytogenet Genome Res.

[27] Kouzarides T (2007). Chromatin modifications and their function. Cell.

[28] Lawrence M, Daujat S, Schneider R (2016). Lateral thinking: how histone modifications regulate gene expression. Trends Genet.

[29] Webber LP, Wagner VP, Curra M (2017). Hypoacetylation of acetyl-histone H3 (H3K9ac) as marker of poor prognosis in oral cancer. Histopathology.

[30] Chen JH, Yeh KT, Yang YM (2013). High expressions of histone methylation- and phosphorylation-related proteins are associated with prognosis of oral squamous cell carcinoma in male population of Taiwan. Med Oncol.

[31] Rinn JL, Chang HY (2012). Genome regulation by long noncoding RNAs. Annu Rev Biochem.

[32] Ponting CP, Oliver PL, Reik W (2009). Evolution and functions of long noncoding RNAs. Cell.

[33] Quinn JJ, Chang HY (2016). Unique features of long non-coding RNA biogenesis and function. Nat Rev Genet.

[34] Pu M, Li C, Qi X (2017). MiR-1254 suppresses HO-1 expression through seed region-dependent silencing and non-seed interaction with TFAP2A transcript to attenuate NSCLC growth. PLoS Genet.

[35] Chen LL (2016). The biogenesis and emerging roles of circular RNAs. Nat Rev Mol Cell Biol.

[36] Zhang M, Huang N, Yang X (2018). A novel protein encoded by the circular form of the SHPRH gene suppresses glioma tumorigenesis. Oncogene.

[37] Huang JZ, Chen M, Ch en (2017). A peptide encoded by a putative lncRNA HOXB-AS3 suppresses colon cancer growth. Mol Cell.

[38] Dimitrova N, Zamudio JR, Jong RM (2014). LincRNA-p21 activates p21 in cis to promote polycomb target gene expression and to enforce the G1/S checkpoint. Mol Cell.

[39] Lin A, Li C, Xing Z (2016). The LINK-A lncRNA activates normoxic HIF1alpha signalling in triple-negative breast cancer. Nat Cell Biol.

[40] Salzman J, Chen RE, Olsen MN (2013). Cell-type specific features of circular RNA expression. PLoS Genet.

[41] Liang D, Wilusz JE (2014). Short intronic repeat sequences facilitate circular RNA production. Genes Dev.

[42] Starke S, Jost I, Rossbach O (2015). Exon circularization requires canonical splice signals. Cell Rep.

[43] Kelly S, Greenman C, Cook PR (2015). Exon Skipping Is Correlated with Exon Circularization. J Mol Biol.

[44] Li Z, Huang C, Bao C (2015). Exon-intron circular RNAs regulate transcription in the nucleus. Nat Struct Mol Biol.

[45] Lauressergues D, Couzigou JM, Clemente HS (2015). Primary transcripts of microRNAs encode regulatory peptides. Nature.

[46] Goldberg AD, Allis CD, Bernstein E (2007). Epigenetics: a landscape takes shape. Cell.

[47] Wang GG, Allis CD, Chi P (2007). Chromatin remodeling and cancer, Part Ⅰ: Covalent histone modifications. Trends Mol Med.

[48] Racki LR, Narlikar GJ (2008). ATP-dependent chromatin remodeling enzymes: two heads are not better, just different. Curr Opin Genet Dev.

[49] Conaway RC, Conaway JW (2009). The INO80 chromatin remodeling complex in transcription, replication and repair. Trends Biochem Sci.

[50] Kwon SY, Grisan V, Jang B (2016). Genome-wide mapping targets of the metazoan chromatin remodeling factor NURF reveals nucleosome remodeling at enhancers, core promoters and gene insulators. PLoS Genet.

[51] Culver-Cochran AE, Chadwick BP (2013). Loss of WSTF results in spontaneous fluctuations of heterochromatin formation and resolution, combined with substantial changes to gene expression. BMC Genomics.

[52] Atsumi Y, Minakawa Y, Ono M (2015). ATM and SIRT6/SNF2H mediate transient H2AX stabilization when dsbs form by blocking HUWE1 to allow efficient gammaH2AX foci formation. Cell Rep.

[53] Erdel F, Rippe K (2011). Chromatin remodelling in mammalian cells by ISWI-type complexes--where, when and why. FEBS J.

[54] Dunaief JL, Strober BE, Guha S (1994). The retinoblastoma protein and BRG1 form a complex and cooperate to induce cell cycle arrest. Cell.

[55] Versteege I, Sevenet N, Lange J (1998). Truncating mutations of hSNF5/INI1 in aggressive paediatric cancer. Nature.

[56] Kadoch C, Hargreaves DC, Hodges C (2013). Proteomic and bioinformatic analysis of mammalian SWI/SNF complexes identifies extensive roles in human malignancy. Nat Genet.

[57] Bao Y, Shen X (2011). SnapShot: Chromatin remodeling: INO80 and SWR1. Cell.

[58] Taberlay PC, Achinger-Kawecka J, Lun AT (2016). Three-dimensional disorganization of the cancer genome occurs coincident with long-range genetic and epigenetic alterations. Genome Res.

[59] Pan Y, AUID- Oho, Liu G (2018). DNA methylation profiles in cancer diagnosis and therapeutics. Clin Exp Med.

[60] Xu P, Hu G, Luo C (2016). DNA methyltransferase inhibitors: an updated patent review (2012-2015). Expert Opin Ther Pat.

[61] Christman JK (2002). 5-Azacytidine and 5-aza-2'-deoxycytidine as inhibitors of DNA methylation: mechanistic studies and their implications for cancer therapy. Oncogene.

[62] Qin T, Jelinek J, Si J (2009). Mechanisms of resistance to 5-aza-2'-deoxycytidine in human cancer cell lines. Blood.

[63] Tsai HC, Li H, Van Neste L (2012). Transient low doses of DNA-demethylating agents exert durable antitumor effects on hematological and epithelial tumor cells. Cancer Cell.

[64] Hollenbach PW, Nguyen AN, Brady H (2010). A comparison of azacitidine and decitabine activities in acute myeloid leukemia cell lines. PLoS One.

[65] Glover AB, Leyland-Jones BR, Chun HG (1987). Azacitidine: 10 years later. Cancer Treat Rep.

[66] Hagemann S, Heil O, Lyko F (2011). Azacytidine and decitabine induce gene-specific and non-random DNA demethylation in human cancer cell lines. PLoS One.

[67] Khan C, Pathe N, Fazal S (2012). Azacitidine in the management of patients with myelodysplastic syndromes. Ther Adv Hematol.

[68] Issa JP, Garcia-Manero G, Giles FJ (2004). Phase 1 study of low-dose prolonged exposure schedules of the hypomethylating agent 5-aza-2'-deoxycytidine (decitabine) in hematopoietic malignancies. Blood.

[69] Kantarjian H, Oki Y, Garcia-Manero G (2007). Results of a randomized study of 3 schedules of low-dose decitabine in higher-risk myelodysplastic syndrome and chronic myelomonocytic leukemia. Blood.

[70] Saunthararajah Y, Sekeres M, Advani A (2015). Evaluation of noncytotoxic DNMT1-depleting therapy in patients with myelodysplastic syndromes. J Clin Invest.

[71] Cowan LA, Talwar S, Yang AS (2010). Will DNA methylation inhibitors work in solid tumors? A review of the clinical experience with azacitidine and decitabine in solid tumors. Epigenomics.

[72] Bellet RE, Catalano RB, Mastrangelo MJ (1978). Phase Ⅱ study of subcutaneously administered 5-azacytidine (NSC-102816) in patients with metastatic malignant melanoma. Med Pediatr Oncol.

[73] Derissen EJ, Beijnen JH, Schellens JH (2013). Concise drug review: azacitidine and decitabine. Oncologist.

[74] Esteller M (2008). Epigenetics in Cancer. N Engl J Med.

[75] Gros C, Fahy J, Halby L (2012). DNA methylation inhibitors in cancer: recent and future approaches. Biochimie.

[76] Zheng YG, Wu J, Chen Z (2008). Chemical regulation of epigenetic modifications: opportunities for new cancer therapy. Med Res Rev.

[77] Kantarjian H, Issa JP, Rosenfeld CS (2006). Decitabine improves patient outcomes in myelodysplastic syndromes: results of a phase Ⅲ randomized study. Cancer.

[78] Schwartsmann G, Schunemann H, Gorini CN (2000). A phase Ⅰ trial of cisplatin plus decitabine, a new DNA-hypomethylating agent, in patients with advanced solid tumors and a follow-up early phase Ⅱ evaluation in patients with inoperable non-small cell lung cancer. Invest New Drugs.

[79] Song SH, Han SW, Bang YJ (2011). Epigenetic-based therapies in cancer: progress to date. Drugs.

[80] Champion C, Guianvarc'h D, Senamaud-Beaufort C (2010). Mechanistic insights on the inhibition of c5 DNA methyltransferases by zebularine. PLoS One.

[81] Jones PA, Taylor SM (1980). Cellular differentiation, cytidine analogs and DNA methylation. Cell.

[82] Silverman LR, Demakos EP, Peterson BL (2002). Randomized controlled trial of azacitidine in patients with the myelodysplastic syndrome: a study of the cancer and leukemia group B. J Clin Oncol.

[83] Issa JJ, Roboz G, Rizzieri D (2015). Safety and tolerability of guadecitabine (SGI-110) in patients with myelodysplastic syndrome and acute myeloid leukaemia: a multicentre, randomised, dose-escalation phase 1 study. Lancet Oncol.

[84] Chuang JC, Warner SL, Vollmer D (2010). S110, a 5-Aza-2'-deoxycytidine-containing dinucleotide, is an effective DNA methylation inhibitor in vivo and can reduce tumor growth. Mol Cancer Ther.

[85] Rilova E, Erdmann A, Gros C (2014). Design, synthesis and biological evaluation of 4-amino-N- (4-aminophenyl)benzamide analogues of quinoline-based SGI-1027 as inhibitors of DNA methylation. Chem Med Chem.

[86] Chen S, Wang Y, Zhou W (2014). Identifying novel selective non-nucleoside DNA methyltransferase 1 inhibitors through docking-based virtual screening. J Med Chem.

[87] Datta J, Ghoshal K, Denny WA (2009). A new class of quinoline-based DNA hypomethylating agents reactivates tumor suppressor genes by blocking DNA methyltransferase 1 activity and inducing its degradation. Cancer Res.

[88] Lam JK, Chow MY, Zhang Y (2015). siRNA versus miRNA as therapeutics for gene silencing. Mol Ther Nucleic Acids.

[89] Li X, Su Y, Sun B (2016). An artificially designed interfering lncRNA expressed by oncolytic adenovirus competitively consumes oncomirs to exert antitumor efficacy in hepatocellular carcinoma. Mol Cancer Ther.

[90] Han M, Jia L, Lv W (2019). Epigenetic enzyme mutations: role in tumorigenesis and molecular inhibitors. Front Oncol.

[91] Rau RE, Rodriguez BA, Luo M (2016). DOT1L as a therapeutic target for the treatment of DNMT3A-mutant acute myeloid leukemia. Blood.

[92] Piekarz RL, Bates SE (2009). Epigenetic modifiers: basic understanding and clinical development. Clin Cancer Res.

[93] Johnstone RW, Licht JD (2003). Histone deacetylase inhibitors in cancer therapy: is transcription the primary target. Cancer Cell.

[94] Bolden JE, Peart MJ, Johnstone RW Anticancer activities of histone deacetylase inhibitors. Nat Rev Drug Discov.

[95] Jones LK, Saha V (2002). Chromatin modification, leukaemia and implications for therapy. Br J Haematol.

[96] Manal M, Chandrasekar MJ, Gomathi Priya J (2016). Inhibitors of histone deacetylase as antitumor agents: A critical review. Bioorg Chem.

[97] Schrump DS (2009). Cytotoxicity mediated by histone deacetylase inhibitors in cancer cells: mechanisms and potential clinical implications. Clin Cancer Res.

[98] Prince HM, Bishton MJ, Harrison SJ (2009). Clinical studies of histone deacetylase inhibitors. Clin Cancer Res.

[99] Rasheed W, Bishton M, Johnstone RW (2008). Histone deacetylase inhibitors in lymphoma and solid malignancies. Expert Rev Anticancer Ther.

[100] Piekarz RL, Frye AR, Wright JJ (2006). Cardiac studies in patients treated with depsipeptide, FK228, in a phase Ⅱ trial for T-cell lymphoma. Clin Cancer Res.

[101] Hu J, Colburn NH (2005). Histone deacetylase inhibition down-regulates cyclin D1 transcription by inhibiting nuclear factor-kappaB/p65 DNA binding. Mol Cancer Res.

[102] Fiskus W, Pranpat M, Bali P (2006). Combined effects of novel tyrosine kinase inhibitor AMN107 and histone deacetylase inhibitor LBH589 against Bcr-Abl-expressing human leukemia cells. Blood.

[103] Scuto A, Kirschbaum M, Kowolik C (2008). The novel histone deacetylase inhibitor, LBH589, induces expression of DNA damage response genes and apoptosis in Ph-acute lymphoblastic leukemia cells. Blood.

[104] Xia C, Leon-Ferre R, Laux D (2014). Treatment of resistant metastatic melanoma using sequential epigenetic therapy (decitabine and panobinostat) combined with chemotherapy (temozolomide). Cancer Chemother Pharmacol.

[105] Kobayashi Y, Ohtsuki M, Murakami T (2006). Histone deacetylase inhibitor FK228 suppresses the Ras-MAP kinase signaling pathway by upregulating Rap1 and induces apoptosis in malignant melanoma. Oncogene.

[106] Bali P, Pranpat M, Bradner J (2005). Inhibition of histone deacetylase 6 acetylates and disrupts the chaperone function of heat shock protein 90: a novel basis for antileukemia activity of histone deacetylase inhibitors. J Biol Chem.

[107] Steele N, Finn P, Brown R (2009). Combined inhibition of DNA methylation and histone acetylation enhances gene re-expression and drug sensitivity in vivo. Br J Cancer.

[108] Knutson SK, Wigle TJ, Warholic NM (2012). A selective inhibitor of EZH2 blocks H3K27 methylation and kills mutant lymphoma cells. Nat Chem Biol.

[109] Teodoridis JM, Hall J, Marsh S (2005). CpG island methylation of DNA damage response genes in advanced ovarian cancer. Cancer Res.

[110] Glasspool RM, Teodoridis JM, Brown R (2006). Epigenetics as a mechanism driving polygenic clinical drug resistance. Br J Cancer.

[111] Konkankit VV, Kim W, Koya RC (2011). Decitabine immunosensitizes human gliomas to NY-ESO-1 specific T lymphocyte targeting through the Fas/Fas ligand pathway. J Transl Med.

[112] Almstedt M, Blagitko-Dorfs N, Duque-Afonso J (2010). The DNA demethylating agent 5-aza-2'-deoxycytidine induces expression of NY-ESO-1 and other cancer/testis antigens in myeloid leukemia cells. Leuk Res.

[113] Dubovsky JA, McNeel DG (2007). Inducible expression of a prostate cancer-testis antigen, SSX-2, following treatment with a DNA methylation inhibitor. Prostate.

[114] Teitell M, Richardson B (2003). DNA methylation in the immune system. Clin Immunol.

[115] Garcia-Manero G, Kantarjian HM, Sanchez-Gonzalez B (2006). Phase 1/2 study of the combination of 5-aza-2'-deoxycytidine with valproic acid in patients with leukemia. Blood.

[116] Gore SD (2005). Combination therapy with DNA methyltransferase inhibitors in hematologic malignancies. Nat Clin Pract Oncol.

[117] Ahrens TD, Timme S, Hoeppner J (2015). Selective inhibition of esophageal cancer cells by combination of HDAC inhibitors and Azacytidine. Epigenetics.

[118] Oki Y, Kantarjian HM, Gharibyan V (2007). Phase Ⅱ study of low-dose decitabine in combination with imatinib mesylate in patients with accelerated or myeloid blastic phase of chronic myelogenous leukemia. Cancer.

[119] Rosato RR, Almenara JA, Yu C (2004). Evidence of a functional role for p21WAF1/CIP1 down-regulation in synergistic antileukemic interactions between the histone deacetylase inhibitor sodium butyrate and flavopiridol. Mol Pharmacol.

[120] Sandor V, Senderowicz A, Mertins S (2000). P21-dependent g(1)arrest with downregulation of cyclin D1 and upregulation of cyclin E by the histone deacetylase inhibitor FR901228. Br J Cancer.

[121] Sjoblom T, Jones S, Wood LD (2006). The consensus coding sequences of human breast and colorectal cancers. Science.

[122] Sekeres MA, Tiu RV, Komrokji R (2012). Phase 2 study of the lenalidomide and azacitidine combination in patients with higher-risk myelodysplastic syndromes. Blood.

[123] Sasidharan Nair V, El Salhat H, Taha RZ (2018). DNA methylation and repressive H3K9 and H3K27 trimethylation in the promoter regions of PD-1, CTLA-4, TIM-3, LAG-3, TIGIT, and PD-L1 genes in human primary breast cancer. Clin Epigenetics.

[124] Almassalha LM, Bauer GM, Wu W (2017). Macrogenomic engineering via modulation of the scaling of chromatin packing density. Nat Biomed Eng.

[125] Lavelle D, Vaitkus K, Ling Y (2012). Effects of tetrahydrouridine on pharmacokinetics and pharmacodynamics of oral decitabine. Blood.

[126] Saunthararajah Y (2013). Key clinical observations after 5-azacytidine and decitabine treatment of myelodysplastic syndromes suggest practical solutions for better outcomes. Hematology Am Soc Hematol Educ Program.

[127] Oki Y, Jelinek J, Shen L (2008). Induction of hypomethylation and molecular response after decitabine therapy in patients with chronic myelomonocytic leukemia. Blood.

[128] Issa JPJ, Kantarjian HM (2009). Targeting DNA methylation. Clin Cancer Res.

[129] Saunthararajah Y, Hillery CA, Lavelle D (2003). Effects of 5-aza-2'-deoxycytidine on fetal hemoglobin levels, red cell adhesion, and hematopoietic differentiation in patients with sickle cell disease. Blood.

[130] Gore SD, Baylin S, Sugar E (2006). Combined DNA methyltransferase and histone deacetylase inhibition in the treatment of myeloid neoplasms. Cancer Res.

[131] Plimack ER, Stewart DJ, Issa JP (2007). Combining epigenetic and cytotoxic therapy in the treatment of solid tumors. J Clin Oncol.

[132] Schrump DS, Fischette MR, Nguyen DM (2006). Phase Ⅰ study of decitabine-mediated gene expression in patients with cancers involving the lungs, esophagus, or pleura. Clin Cancer Res.

[133] Samlowski WE, Leachman SA, Wade M (2005). Evaluation of a 7-day continuous intravenous infusion of decitabine: inhibition of promoter-specific and global genomic DNA methylation. J Clin Oncol.

[134] Topper MJ, Vaz M, Chiappinelli KB (2017). Epigenetic therapy ties MYC depletion to reversing immune evasion and treating lung cancer. Cell.

